# QualityRights in medical education to reduce coercion in mental health in Colombia: instrument validation and quasi-experimental study

**DOI:** 10.1192/bjo.2026.11042

**Published:** 2026-05-11

**Authors:** Felipe Agudelo-Hernández, Helena Vélez-Botero, Andrés Camilo Delgado-Reyes

**Affiliations:** Department of Mental Health, Caldas Territorial Health Office, Manizales, Colombia; Department of Psychology, University National of Colombia, Bogotá, Colombia; Department of Psychology, University of Manizales, Manizales, Colombia

**Keywords:** Mental health, coercion, education, mental health services, validation study

## Abstract

**Background:**

The QualityRights initiative has shown benefits in decreasing coercive practices and enhancing the recognition of human rights in healthcare.

**Aims:**

To translate the World Health Organization’s (WHO) QualityRights Practices Questionnaire into Spanish and assess the relationships between coercive practices in healthcare settings, perceptions of mental disorders, and attitudes and commitment to human rights among primary healthcare professionals from multiple disciplines. Also, we sought to compare these variables between trained and untrained professionals, and evaluate longitudinal outcomes of integrating QualityRights training into undergraduate medical education.

**Method:**

A quasi-experimental study with a non-equivalent control group was conducted. Instruments included the Community Attitudes Toward the Mentally III, Human Rights Exposure in Social Work and Human Rights Engagement in Social Work scales, and the WHO QualityRights Practices Questionnaire. A total of 260 professionals from 12 Colombian regions participated in the study. Multiple linear regression analyses were conducted to examine the effects of the QualityRights initiative on various dimensions related to human rights.

**Results:**

Translation and validation of the WHO QualityRights Practices Questionnaire yielded excellent psychometric properties (Kaiser–Meyer–Olkin value: 0.945; Cronbach’s *α* = 0.891–0.923; single component explaining 73.4% of variance). The QualityRights initiative was significantly associated with greater human rights knowledge and lower endorsement of coercive practices and authoritarian beliefs. In relation to coercive practices, the initiative was significantly associated with lower scores on the QualityRights Practices Questionnaire (*B* = −3.118, *p* < 0.001).

**Conclusions:**

In Colombia, incorporating the QualityRights initiative into medical education appears to be a promising strategy for reducing stigma, enhancing knowledge and commitment to human rights, and minimising coercive practices in primary mental healthcare.

Globally, the prevalence and burden of mortality associated with mental illness have increased significantly. However, the COVID-19 pandemic further exacerbated this situation, leading to a rise in mental health-related hospital admissions.^1^ Currently, many people live with health problems, and 80% of mental health budgets in low- and middle-income countries are allocated to mental hospitals.^2^ In several Latin American countries, the primary containment mechanism remains hospital-based services with asylum-like characteristics,^2,4,18^ where coercive practices that violate human rights prevail – including in Colombia.^4^

Coercive practices refer to actions by healthcare personnel that include restrictive measures such as seclusion and physical restraint (also known as manual), mechanical and chemical restraint, and involuntary admission, which occurs without the person’s consent.^5^ The study by Savage et al,^5^ which collected data from 2013 to 2022 on involuntary hospital admissions, seclusion, mechanical and physical restraint, and involuntary medication in various European countries and the USA, highlights that these highly prevalent practices are both psychologically and physically harmful, sometimes resulting in death. The authors also note that alternatives to coercive practices are cost-effective, lead to fewer injuries and result in quicker resolution of crises.

Current studies show wide variability in the prevalence of coercive practices across countries, reflecting cultural, economic and political differences.^6^ Physical/mechanical restraint ranges from 0.3% in the USA to 54% in Italy. Seclusion rates range from 0.2% in the USA to 56% in Japan, while chemical restraint varies from 2% in Norway to 58% in India. These differences suggest that coercive practices are shaped by local regulations, staff training and available resources.^7^

The international human rights framework in mental health is primarily defined by the Convention on the Rights of Persons with Disabilities (CRPD), which establishes key principles such as non-discrimination, prohibition of cruel or degrading treatment, and the requirement for free and informed consent. Over the past few decades, three major transformations have taken place in the field of mental health care: (a) deinstitutionalisation, (b) the adoption of the recovery model and (c) the enforcement of the CRPD.^6,8^ Each of these has required significant legislative reform to actively promote the conditions necessary for all individuals to fully realise their rights.

Some countries have adopted the Convention’s guidelines by reforming their public policies, including Scotland,^9^ The Netherlands^10^ and Australia.^11^ In Latin America, legal reforms have been enacted to align with the CRPD in several countries, including Chile (Law 21.331), Peru (Law No. 30947), Argentina (Law 26657), Brazil (Law 1.216), Uruguay (Decree 305/011), Panama (Law 364), Mexico (General Health Law), Nicaragua (Law No. 650) and Colombia, which enacted Law 1616 of 2013 to guarantee the right to mental health – although specific standards on coercive practices remain limited.^12^

The World Health Organization (WHO) acknowledges that, because of the limitations of current mental health systems and the complexity of care contexts, involuntary practices may still occur, even when personnel make substantial efforts to implement alternatives to coercion. Therefore, key areas of action must be addressed, including funding, research into strategies to prevent coercive practices, and training and education.^8^

Regarding the latter, human rights education encompasses all educational, training, informational, awareness-raising and learning activities aimed at promoting universal respect for, and enjoyment of, all human rights and fundamental freedoms.^13^ This contributes to preventing human rights violations and abuses by equipping both patients and health professionals with knowledge, skills and understanding, while shaping attitudes and behaviours that empower them to promote a universal culture of human rights.^14^

Among the various professions involved in healthcare, such as social workers, psychologists and nurses, social workers have most effectively integrated the importance of human rights into both their practice and academic curricula.^15,20^ The goal of human rights education is to foster a culture of respect for others, acknowledging all recognised differences. By learning about human rights, health professionals can enhance medical practice worldwide by promoting more ethical, equitable and effective healthcare.^13,14^

Individual and institutional barriers limit the effectiveness of these policy advances. Barriers to addressing human rights violations in healthcare services include a general lack of knowledge about human rights among the public and mental health professionals, as well as negative attitudes toward individuals with psychosocial disabilities, which contribute to coercive practices in mental health services.^16^ It has been proposed that greater knowledge of human rights and stronger commitment among mental health professionals and other stakeholders to implementing a rights-based framework would lead to positive changes in attitudes toward individuals with mental health conditions or psychosocial disabilities as rights holders, in addition to reducing coercive practices.^6,8^

In terms of political prioritisation, international conventions and treaties influence national legislation in various countries, helping to address the care and social needs of individuals with mental health conditions. The QualityRights initiative has shown benefits in reducing coercive practices and in promoting the recognition of human rights in mental health contexts.^8,17,21^

This is considered a positive step toward reducing stigma and increasing inclusion in employment, education and academic settings.^8^ A recent review study that compiled implementation processes and progress made with QualityRights up to 2022 identified three key areas for improving the effectiveness of the strategy: (a) political prioritisation, (b) system-level funding for change and (c) promotion of feasible and contextually adapted measures to support accountability and programme refinement.^21^

In Colombia, recent legislative advances include the enactment of the Mental Health Law (Law 2460 of 2025), which reinforces the right to mental health care through community-based services and explicitly prohibits coercive practices. This law builds upon previous frameworks such as Law 1616 of 2013 and the Statutory Health Law (Law 1751 of 2015), which recognise health as a fundamental right. However, significant implementation challenges persist because of weak intersectoral coordination, insufficient provider training and the absence of robust monitoring mechanisms.^18^ These structural issues often result in limited application of the human rights framework in real-world clinical practice.^18^

Despite these advances, the systematic monitoring of coercive practices requires validated instruments. Current tools vary considerably in scope and psychometric properties, and few have been culturally adapted for Latin American healthcare contexts, limiting the comparability of findings and the evaluation of training interventions.

## The present study

Previous research in similar contexts has highlighted the impact of the QualityRights initiative in reducing stigma and improving knowledge and commitment to human rights.^4^ However, there is a lack of validated and culturally adapted instruments for assessing coercive practices in health services and substitute decision-making. Despite the global recommendation for the implementation of this strategy, few studies have evaluated its effects within Latin American settings. Although several WHO QualityRights training and guidance materials are officially available in Spanish, an official Spanish version of the WHO QualityRights Practices Questionnaire was not identified in the WHO sources consulted. Therefore, for this study, the instrument was translated into Spanish and culturally adapted before administration. A cultural adaptation was therefore necessary to verify terminological comprehension, item functioning and structural validity in this specific context.

The present study aimed to (a) translate and assess the psychometric properties of the WHO QualityRights Practices Questionnaire in the Colombian context; (b) examine associations between coercive practices, human rights knowledge and perceptions of mental illness among primary healthcare professionals; (c) compare these variables between QualityRights-trained and untrained professionals; and (d) evaluate longitudinal outcomes of integrating QualityRights training into undergraduate medical education. We hypothesised that healthcare professionals who received QualityRights training would demonstrate more positive perceptions of individuals with mental disorders, greater awareness and commitment to human rights, and reduced use of coercive practices in their clinical practice. We also hypothesised that the use of coercive practices would be associated with limited knowledge and commitment to human rights, as well as with negative perceptions of individuals with mental illness.

## Method

### Study design

This quasi-experimental study used a non-equivalent control group design. Three measurement phases were conducted. The first phase consisted of administering scales to 194 students before beginning the QualityRights training, serving as a baseline measurement. However, because the comparison group was not assessed at this same time point, a formal pre–post experimental design was not established; this limits the ability to attribute observed changes exclusively to the intervention. The second phase consisted of administering the instruments after the training, to the same 194 students.

The third phase was 6 months after the training, when these students were already professionals and were working in primary health care. For this third phase, professionals who had received specific training in QualityRights were compared with those who had not, without random assignment, within the framework of a structured educational intervention. Eighty-two professionals were contacted for the third phase of the measurement, compared with 178 professionals who did not receive this training from multiple regions of Colombia. The remaining professionals were unable to participate in the third phase because they were not working in primary care or mental health services, or because we were unable to contact them.

### Participants and procedure

A convenience sampling strategy was used, based on the researchers’ ability to train and monitor a group of professionals. The trained group consisted of final-year medical students (*n* = 82 at phase 3) who completed QualityRights training as part of their psychiatry course during the academic year preceding the main data collection phase; phases 1 and 2 (pre- and post-training assessments) were collected during that same academic year.

The training was proposed in the community mental health course as a supplement to the training, as it was not planned in the medical programme curriculum. Non-participation had no impact on the students’ seminar grade. It is acknowledged, however, that as training completion was a course requirement, the educational context may have provided implicit incentives for study participation, which should be considered when interpreting self-reported outcomes. None of the participants in the trained group had received QualityRights training before the academic period in which this study was conducted.

Because of logistical constraints and the decentralised structure of healthcare training and services in Colombia, a convenience sampling strategy was employed, followed by snowball sampling to increase reach among professionals in different regions. Although this approach may introduce selection bias, particularly regarding age and professional experience, efforts were made to balance the sample across institutions, territories and disciplines.

The comparison group was composed of primary care professionals actively working in the Colombian health system who had not received QualityRights training. This group was recruited via snowball sampling by digitally disseminating the survey through regional mental health administrators. A key exclusion criterion for this group was prior participation in the QualityRights programme. Following the study, these professionals were encouraged to complete the virtual QualityRights training.

The experimental group (*n* = 82) consisted of recent medical graduates with limited professional experience, whereas the control group (*n* = 178) included practising professionals from diverse disciplines with varying levels of work experience. This difference represents a potential confounding variable that was not controlled through matching. To address this limitation analytically, profession, age and region were included as covariates in the multiple regression models.

The training itself was publicly available, self-guided and delivered through a virtual platform, in Spanish, with support from a psychiatry instructor during the final semester of medical training (Supplementary File 1). Additionally, trainees were accompanied by a person with lived experience of coercive practices. Completion of the training was a course requirement, although participation in the study was not mandatory (https://www.who.int/teams/mental-health-and-substance-use/policy-law-rights/qr-e-training).

The training began with awareness-raising on the Declaration of Human Rights, CRPD and the stigma towards people with mental disorders. It was followed by the ‘Mental Health, Disability, and Human Rights’ module, the ‘Legal Capacity and the Right to Decide’ module and the ‘Ending Coercion, Violence, and Abuse’ module. Strategies for eliminating coercive practices in health services were described. The ‘Quality Services and Community Inclusion’ and ‘Mental Health, Well-being, and Recovery’ modules were also covered. This training took place over 6 weeks, with one session per week.

### Instruments

Sociodemographic variables collected included gender, age (years), profession (medicine, psychology, social work, nursing) and region of practice were collected by *ad hoc* instrument. All instruments were administered in Spanish.

### Community Attitudes Toward the Mentally III scale

The Community Attitudes Toward the Mentally III (CAMI) scale assesses the level of acceptance or rejection toward people with mental illness and explains community reactions to mental health service settings. It has been validated in various contexts, including Colombia.^18^ The scale comprises four subscales: authoritarianism, benevolence, social restrictiveness and mental health ideology (20 items total; subscale score range: 10–50; no validated cut-off scores available for the Colombian version). Each subscale includes five positively and five negatively worded statements, rated on a five-point Likert scale from ‘strongly agree’ to ‘strongly disagree’. Initial internal consistency ranged from *α* = 0.68 to 0.88, whereas the Colombian version reported values between 0.59 and 0.80.^19^ In our sample, the internal consistency of the CAMI scale was high (Cronbach’s *α* = 0.887).

### Human Rights Exposure in Social Work and Human Rights Engagement in Social Work scales

The Human Rights Exposure in Social Work (HRXSW) scale measures knowledge and exposure to human rights principles, whereas the Human Rights Engagement in Social Work (HRESW) assesses the understanding of professional practice within a human rights framework. Both scales have been used in Latin American studies.^4^ The HRXSW includes 11 items; the HRESW includes 25 items across 3 domains: support for human rights principles, perceived relevance to practice and application in professional settings. Responses are scored on a seven-point Likert scale. In its Spanish validation, the HRXSW included 16 items and the HRESW retained 25 items, yielding Cronbach’s *α* values of 0.803 and 0.856, respectively.^20^ The HRXSW scale (Cronbach’s *α* = 0.949) and HRESW (Cronbach’s *α* = 0.948) scale also showed excellent internal consistency in our sample, and no clinical cut-off scores established.

### WHO QualityRights Practices Questionnaire: coercion subscale

This instrument includes two subscales focused on coercive strategies such as seclusion, physical restraint, forced medication, chemical restraint and verbal aggression. The first subscale (QRA) assesses the respondent’s use of such strategies in the past 3 months, using a Likert scale ranging from ‘never’ to ‘every day’. The second subscale (QRB) measures the respondent’s perception of how often other health professionals at their institution employ these practices, rated from ‘much less than I do’ to ‘much more than I do’. Moro et al^16^ reported a high content validity index (0.976), and a confirmatory factor analysis supported a final nine-item model with acceptable fit (root mean square error of approximation = 0.070, comparative fit index = 0.99, Tucker–Lewis index = 0.99). Cronbach’s *α* for the full scale was 0.75 (subscale 1 = 0.74; subscale 2 = 0.89), indicating good reliability.

The coercion scale was translated into Spanish by the research team, who developed a preliminary version through consensus. Discrepancies related to synonyms, uncommon terms, phrase structure or complexity were addressed, and a final version was agreed on. A professional translator then performed both a back-translation (Spanish to English) and a new forward translation (English to Spanish). These translations were compared to the original instrument, revealing no major semantic differences.

For face and content validity, three experts in social psychiatry, health psychology and social work, with experience in coercive practices and knowledge of QualityRights, reviewed the items with an online form. Ten criteria were assessed: clarity, objectivity, relevance, organisation, sufficiency, adequacy, consistency, coherence, methodology and significance, along with linguistic observations. Aiken’s *V* was used to compute the content validity coefficient, with a threshold of ≥0.80 for item adequacy, which all items met.

A semi-structured cognitive interview was conducted using the final version (Supplementary File 2). This aimed to assess respondents’ comprehension, recall and response formulation. The goal was to detect and address potential sources of error, thus enhancing the instrument’s quality and effectiveness. Six primary care professionals from six Colombian departments participated. Overall, items were found to be understandable; some participants reported initial discomfort, which diminished once confidentiality was assured.

### Statistical analysis

To evaluate the psychometric properties of the WHO QualityRights Practices Questionnaire, a principal component analysis with Varimax rotation was conducted. Internal consistency for each subscale was calculated with Cronbach’s *α*. This adaptation was conducted to ensure contextual and terminological suitability for Colombian healthcare professionals, following a forward–back translation protocol, given that the WHO version was developed for a broader international audience and required local validation.

To explore the relationships between coercive practices, human rights knowledge and commitment, and perceptions of mental illness, Spearman’s rank-order correlations were computed because the Kolmogorov–Smirnov test indicated non-normal distributions for all variables. To compare attitudinal and behavioural differences between professionals who received QualityRights training and those who did not, Mann–Whitney *U*-tests were performed on each dependent variable, with *z*-statistics reported. Effect sizes were calculated using Cohen’s *r* (*r* = *z*/√*N*) and interpreted as small (0.10), moderate (0.30) or large (approximately 0.50), following Cohen’s guidelines. For phase 1 pre–post comparisons (within the trained group), Wilcoxon signed-rank tests were used.

For the longitudinal subsample of 82 physicians assessed at three time points, Shapiro–Wilk tests indicated non-normal distributions, so non-parametric procedures were used. A Friedman test evaluated overall changes across pre-training, post-training and follow-up. When significant, Wilcoxon signed-rank tests were applied for pairwise comparisons. To reduce type 1 error, a Bonferroni adjustment set the significance threshold at *α* = 0.017. Effect sizes were calculated and interpreted as small (0.10), moderate (0.30) or large (approximately 0.50), following Cohen’s guidelines.

To identify predictors of human rights knowledge, commitment and attitudes, multiple linear regression models were built for each dependent variable. Independent variables included training, profession, gender, age and region. These variables were included as categorical predictors and coded numerically in SPSS. The Durbin–Watson statistic was used to assess residual autocorrelation in all models. All analyses were conducted using SPSS version 26 for Windows (IBM Corporation, Armonk, New York; https://www.ibm.com/mx-es/products/spss-statistics), and statistical significance was set at *p* < 0.05 (two-tailed).

### Ethics approval and consent to participate

The authors assert that all procedures contributing to this work comply with the ethical standards of the relevant national and institutional committees on human experimentation and with the Helsinki Declaration of 1975, as revised in 2013. All procedures involving human patients were approved by the Ethics Committee of the University of Manizales through act CB04-2024.

Absolute confidentiality of the names of the study participants was guaranteed, and participants provided their consent to participate. Each participant received an explanation of the methodology and sufficient time was provided to address any concerns from the participants. Informed consent was obtained from all individual participants included in the study. Verbal consent was witnessed and formally recorded.

## Results

No missing data or multivariate outliers were identified in either the phase 1 (*n* = 194) or phase 2 (*n* = 260) samples. Results are organised as follows: (a) psychometric properties of the WHO QualityRights Practices Questionnaire, (b) sociodemographic descriptives, (c) associations and group comparisons (phase 2) and (d) longitudinal analyses (phase 3 subsample).

### Psychometric properties of the WHO QualityRights Practices Questionnaire

The translation and adaptation procedures for the WHO QualityRights Practices Questionnaire are described in the section ‘WHO QualityRights Practices Questionnaire (QR Practices): Coercion Subscale’. Psychometric results are reported below. To assess the internal structure of the WHO QualityRights Practices Questionnaire, we conducted a principal component analysis with Varimax rotation. The Kaiser–Meyer–Olkin (KMO) measure of sampling adequacy was 0.945, indicating excellent suitability for factor analysis, and Bartlett’s test of sphericity was significant (*χ* ^2^ = 2481.83, d.f. = 36, *p* < 0.001). One dominant component explained 73.4% of the total variance. Factor loadings ranged from 0.403 to 0.956, with QRB items loading negatively and QRA items loading positively, confirming the internal coherence of the two subscales.

Reliability was also assessed using Cronbach’s *α*. The internal consistency for the perception subscale (QRB) was *α* = 0.923 across four items, and for the self-reported practices subscale (QRA), *α* = 0.891 across five items. These values indicate excellent reliability for both subscales within the Colombian context. As evidence of convergent validity, significant correlations were observed between the WHO QualityRights Practices Questionnaire subscales and theoretically related constructs such as authoritarianism, benevolence and mental health ideology (see Table 1), supporting the instrument’s conceptual alignment. The scale was validated with 260 health professionals.

**Table 1:** Spearman rank-order correlations between study variables, phase 2 full sample (*n* = 260)

Spearman correlation	HRXSW notions	HRESW commitment	QRA	QRB	CAMI authoritarianism	CAMI benevolence	CAMI social restriction	CAMI mental health ideology
QualityRights training (coefficient correlation) (*r*)	0.281**	0.011	−0.808**	0.808**	−0.165**	−0.156*	−0.061	−0.049
Significance	<0.001	0.864	<0.001	<0.001	0.008	0.012	0.330	0.430
Territory (coefficient correlation) (*r*)	−0.253**	−0.063	0.657**	−0.648**	0.072	0.029	−0.042	<0.001
Significance	<0.001	0.309	<0.001	<0.001	0.244	0.643	0.505	0.994
Profession (coefficient correlation) (*r*)	0.152*	0.045	−0.537**	0.647**	0.240**	0.155*	0.267**	0.150*
Significance	0.014	0.475	<0.001	<0.001	<0.001	0.012	<0.001	0.016
Gender (coefficient correlation) (*r*)	−0.064	−0.043	0.114	−0.070	0.015	0.088	0.071	0.075
Significance	0.303	0.493	0.067	0.259	0.806	0.158	0.255	0.231
Age (coefficient correlation) (*r*)	−0.137*	0.031	0.513**	−0.562**	0.161**	0.105	0.020	0.073
Significance	0.028	0.617	<0.001	<0.001	0.009	0.092	0.751	0.243
HRXSW notions (coefficient correlation) (*r*)		0.398**	−0.147*	0.135*	−0.049	−0.057	−0.110	0.141*
Significance		<0.001	0.018	0.029	0.433	0.359	0.077	0.023
HRESW commitment (coefficient correlation) (*r*)			0.018	0.013	0.048	0.227**	−0.033	0.089
Significance			0.777	0.835	0.437	<0.001	0.595	0.153
QRA (coefficient correlation) (*r*)				−0.648**	0.185**	0.187**	0.116	0.085
Significance				<0.001	0.003	0.002	0.062	0.173
QRB (coefficient correlation) (*r*)					−0.178**	−0.065	0.051	−0.079
Significance					0.004	0.296	0.414	0.202
CAMI authoritarianism (coefficient correlation) (*r*)						0.504**	0.528**	0.474**
Significance						<0.001	<0.001	<0.001
CAMI benevolence (coefficient correlation) (*r*)							0.523**	0.380**
Significance							<0.001	<0.001
CAMI social restriction (coefficient correlation) (*r*)								0.339**
Significance								<0.001

HRXSW, Human Rights Exposure in Social Work scale; HRESW, Human Rights Engagement in Social Work scale; QRA, World Health Organization’s QualityRights Practices Questionnaire, subscale A; QRB, World Health Organization’s QualityRights Practices Questionnaire, subscale B; CAMI, Community Attitudes Toward the Mentally III scale. Significance (two-tailed): *p* < 0.01(**)/*p* < 0.05(*).

Item-total correlations were examined for both subscales. For the QRA subscale (five items), correlations with the subscale total ranged from *r* = 0.552 (QRA item 1) to *r* = 0.927 (QRA item 5). For the QRB subscale (four items), correlations ranged from *r* = 0.854 (QRB item 4) to *r* = 0.964 (QRB item 2). All items substantially exceeded the recommended threshold of 0.30, confirming adequate item contribution to each subscale.

For the second follow-up, a total of 260 professionals from 12 Colombian territories participated (mean age: 29.3 years, s.d. = 6.7); 71.2% were female. Table 2 summarises participants’ sociodemographic composition by QualityRights training status (no/yes) and overall. Medicine was the largest professional group (41.9% of the total sample), followed by psychology (23.8%), social work (19.2%) and nursing (15.0%). Geographically, Caldas contributed the largest share of participants (34.6%), driven by the concentration of trained participants in that territory (all trained participants were located in Caldas). Other notable territories included Arauca (13.1%), Barranquilla (11.9%), Amazonas (7.7%) and Santander (7.7%), whereas participation from Cali and Guaviare was minimal (0.4 and 2.3%, respectively). Table 3 also reports age distribution: participants ranged from 22 to 54 years (mean 29.3 years, s.d. = 6.7); 14% were younger than 25 years, 62% were between 25 and 35 years and 24% were older than 35 years. This age pattern illustrates the heterogeneity of the non-trained subgroup (*n* = 178) in contrast to the more homogeneous profile of the recently graduated, QualityRights-trained cohort.

**Table 2:** Sociodemographic characteristics

		QualityRights training		Total (%)
Sociodemographic variables		No (%)	Yes (%)	
Gender	Female	69.1	75.6	71.2
	Male	30.9	24.4	28.8
Age	<25 years	5.7	14.6	8.6
	25–35 years	86.5	85.4	86.0
	>35 years	8.1	0.0	5.4
Territory	Caldas	4.5	100.0	34.6
	Risaralda	10.7		7.3
	Casanare	7.3		5.0
	Santander	11.2		7.7
	Chocó	9.6		6.5
	Cali	0.6		0.4
	Guaviare	3.4		2.3
	Meta	5.1		3.5
	Barranquilla	17.4		11.9
	Amazonas	11.2		7.7
	Arauca	19.1		13.1
Profession	Social work	28.1		19.2
	Psychology	34.8		23.8
	Nursing	21.9		15.0
	Medicine	15.2	100.0	41.9

Full sample (*N* = 260) by QualityRights training status (trained: *n* = 82; untrained: *n* = 178).

**Table 3:** Pre–post comparison of study variables, first follow-up

Comparison	Pre-test		Post-test		*z*-value	Significance	Cohen’s *r*
	Mean	s.d.	Mean	s.d.			
HRXSW notions (post-test − pre-test)	4.33	1.37	4.82	1.27	−3.770	<0.001	0.270
HRESW commitment (post-test − pre-test)	5.61	1.06	5.55	1.07	−0.574	0.567	0.040
CAMI authoritarianism (post-test − pre-test)	3.01	0.57	2.78	0.48	−3.807	<0.001	0.272
CAMI benevolence (post-test − pre-test)	3.03	0.53	2.88	0.40	−3.110	<0.001	0.224
CAMI social restrictiveness (post-test − pre-test)	2.87	0.57	2.75	0.41	−1.334	0.181	0.095
CAMI mental health ideology (post-test − pre-test)	3.10	0.53	2.95	0.41	−2.748	0.005	0.198

*n* = 194. HRXSW, Human Rights Exposure in Social Work scale; HRESW, Human Rights Engagement in Social Work scale; CAMI, Community Attitudes Toward the Mentally III scale.

Cohen’s *r*: effect size estimated from *z*-statistic (*r* = *z*/√*N*). Small = 0.10; moderate = 0.30; large = 0.50.

### Sociodemographic descriptives

At the first follow-up (student group), which initially consisted of 194 students, a comparison of the results after training showed changes such as increased notions of human rights, decreased stigmatising negative attitudes toward mental health, and lower scores on the authoritarianism and benevolence components (Table 3).

In addition to these initial findings, effect size analyses indicated small-to-moderate improvements across domains, particularly in human rights notions (*r* = 0.27) and authoritarianism (*r* = 0.27), supporting the relevance of the training in modifying attitudes during undergraduate education. These results highlight that the QualityRights training not only produced statistically significant changes, but also meaningful effects on specific attitudinal dimensions, reinforcing its potential value as an educational intervention.

### Associations and group comparisons

The statistical analyses confirmed consistent associations between QualityRights training and key outcomes. Specifically, training participation was positively correlated with human rights notions (*ρ* = 0.281, *p* < 0.01) and negatively correlated with authoritarian beliefs (*ρ* = –0.165, *p* < 0.01). Profession and territory also showed significant correlations with authoritarianism, benevolence, social restriction and mental health ideology, suggesting that both professional background and geographic context influenced attitudinal profiles (Table 1). These findings provide quantitative support for the role of QualityRights training in shaping attitudes and perceptions, without relying on anecdotal or selective feedback.

Additionally, the scales of authoritarianism, benevolence, social restriction and mental health ideology were significantly and positively interrelated (e.g. authoritarianism and benevolence (*ρ* = 0.504, *p* < 0.01). These results suggest complex associations between sociodemographic factors and attitudinal variables, particularly in relation to professional training and territorial context (Table 1).

To provide a clearer picture of the diversity of the non-trained group, descriptive analyses were conducted separately for the 178 professionals who did not receive QualityRights training. These data are presented without direct comparison to the 82 recently graduated physicians, acknowledging the methodological limitations of comparing such heterogeneous groups. Instead, the descriptive statistics illustrate the variability in attitudes and practices within this broader sample of practitioners, highlighting the contextual heterogeneity across disciplines and territories.

Additionally, a significant decrease was observed in the scores of coercive practices on both subscales QRA and QRB, indicating that the training helped reduce the acceptance or implementation of coercive measures in the mental health field. Lower scores in authoritarianism and benevolence were also observed in the trained group, which could be interpreted as a reduction in paternalistic or condescending attitudes, as well as authoritarian tendencies in interactions with people experiencing psychological distress. In contrast, no statistically significant differences were found in the variables of human rights commitment, social restriction or mental health ideology, suggesting these aspects were not significantly altered by the intervention (Table 4).

**Table 4:** QualityRights training effects

Variables	Friedman mean rank		Wilcoxon *W* comparisons	*z*-value	*p*-value	*r* (effect size)
HRXSW notions	Pre	12.00	Pre versus post	−3.32	0.00	0.37
	Post	14.04	Post versus follow-up	−0.38	0.71	0.04
	Follow-up	14.01	Pre versus follow-up	−3.29	0.00	0.36
HRESW commitment	Pre	15.11	Pre versus post	−0.94	0.35	0.10
	Post	15.79	Post versus follow-up	−0.07	0.94	0.01
	Follow-up	15.76	Pre versus follow-up	−0.98	0.33	0.11
CAMI authoritarianism	Pre	7.73	Pre versus post	−1.81	0.07	0.20
	Post	6.16	Post versus follow-up	0.00	1.00	0.00
	Follow-up	6.16	Pre versus follow-up	−1.81	0.07	0.20
CAMI benevolence	Pre	7.26	Pre versus post	−0.70	0.48	0.08
	Post	6.98	Post versus follow-up	0.00	1.00	0.00
	Follow-up	6.98	Pre versus follow-up	−0.70	0.48	0.08
CAMI social restriction	Pre	5.49	Pre versus post	−0.31	0.76	0.03
	Post	5.60	Post versus follow-up	0.00	1.00	0.00
	Follow-up	5.60	Pre versus follow-up	−0.31	0.76	0.03
CAMI mental health ideology	Pre	8.91	Pre versus post	−0.80	0.43	0.09
	Post	8.70	Post versus follow-up	0.00	1.00	0.00
	Follow-up	8.70	Pre versus follow-up	−0.80	0.43	0.09

HRXSW, Human Rights Exposure in Social Work scale; HRESW, Human Rights Engagement in Social Work scale; CAMI, Community Attitudes Toward the Mentally III scale.

### Longitudinal analyses

A longitudinal comparison of the 82 medical graduates who were followed up on after entering primary care practice showed that improvements observed immediately post-training were partially sustained. A Friedman test was conducted to examine changes across the three assessment points (pre-training, immediate post-training and follow-up) on all six subscales. The omnibus test revealed a highly significant overall difference across time (*χ* ^2^(35) = 2592.95, *p* < 0.001), indicating that at least some of the subscales changed significantly over time. *Post hoc* pairwise Wilcoxon tests were therefore conducted for each subscale to identify where the differences occurred (Table 4).

*Post hoc* Wilcoxon signed-rank tests with a Bonferroni-adjusted *α* of 0.017 (0.05/3 comparisons) revealed that the HRXSW notions subscale showed the most consistent gains: scores increased significantly from pre- to post-training (*Z* = –3.32, *p* = 0.001, *r* = 0.37; moderate effect), and these gains were largely sustained at follow-up (pre-training to follow-up: *Z* = –3.29, *p* = 0.001, *r* = 0.36; moderate effect). For the HRESW commitment subscale, changes were in the expected direction – higher commitment scores post-training and at follow-up – but did not reach the Bonferroni-adjusted significance threshold (pre- to post-training: *Z* = –0.94, *p* = 0.346, *r* = 0.10; small effect).

The attitudinal subscales also showed small reductions in authoritarian and restrictive attitudes or slight improvements in benevolence; however, all effect sizes were small or negligible (*r* ≤ 0.20), and none were statistically significant after correction (all *p*-values > 0.017).

Finally, multiple linear regression analyses were conducted to examine the effects of QualityRights training and sociodemographic variables on various dimensions related to human rights, social beliefs and ideological attitudes (Table 5). Regarding the HRXSW notions subscale, QualityRights training had a significant positive effect (*B* = 0.691, *p* = 0.027), suggesting that receiving this training is associated with greater awareness or knowledge about human rights. No other predictor reached significance in this model, although the model was statistically significant overall (*F*(5, 253) = 4.361, *p* = 0.001), with an adjusted *R* ^2^ of 6.1%.

**Table 5:** Comparison of scale scores according to participation in QualityRights training

Dependent variable	Model	Unstandardised coefficients		Standardised coefficients	*t*	Significance	Model summary				
		*B*	s.d.	*β*			*r*	*r* ^2^	Adjusted *r* ^2^	s.e. of the estimate	Durbin–Watson statistic
HRXSW notions	(Constant)	4.443	0.932		4.768	<0.001	0.282	0.079	0.061	1.287	1.899
	QualityRights training	0.691	0.310	0.243	2.232	0.027					
	Territory	−0.039	0.025	−0.128	−1.530	0.127					
	Profession	−0.141	0.096	−0.125	−1.470	0.143					
	Gender	−0.244	0.177	−0.083	−1.376	0.170					
	Age	0.004	0.022	0.014	0.197	0.844					
HRESW commitment	(Constant)	6.229	0.791		7.879	<0.001	0.177	0.031	0.012	1.09205	2.239
	QualityRights training	−0.232	0.263	−0.098	−0.883	0.378					
	Territory	−0.041	0.021	−0.164	−1.921	0.056					
	Profession	0.020	0.082	0.022	0.248	0.804					
	Gender	−0.305	0.150	−0.126	−2.028	0.044					
	Age	0.007	0.019	0.026	0.349	0.727					
QRA	(Constant)	7.228	0.330		21.882	<0.001	0.951	0.904	0.902	0.45630	1.896
	QualityRights training	−3.118	0.110	−0.995	−28.397	<0.001					
	Territory	0.005	0.009	0.014	0.534	0.594					
	Profession	0.107	0.034	0.086	3.137	0.002					
	Gender	0.126	0.063	0.039	2.013	0.045					
	Age	0.000	0.008	0.001	0.048	0.962					
QRB	(Constant)	0.014	0.276		0.051	0.959	0.948	0.899	0.897	0.38072	1.819
	QualityRights training	2.213	0.092	0.870	24.148	<0.001					
	Territory	−0.005	0.007	−0.018	−0.645	0.519					
	Profession	0.057	0.028	0.057	2.019	0.045					
	Gender	−0.023	0.052	−0.009	−0.443	0.658					
	Age	−0.012	0.007	−0.045	−1.859	0.064					
CAMI authoritarianism	(Constant)	2.711	0.344		7.876	<0.001	0.550	0.303	0.289	0.4754	1.816
	QualityRights training	−0.853	0.114	−0.705	−7.459	<0.001					
	Territory	−0.016	0.009	−0.122	−1.678	0.095					
	Profession	0.339	0.035	0.707	9.549	<0.001					
	Gender	0.045	0.065	0.037	0.695	0.488					
	Age	0.003	0.008	0.027	0.423	0.673					
CAMI benevolence	(Constant)	2.950	0.326		9.043	<0.001	0.459	0.211	0.195	0.4505	1.977
	QualityRights training	−0.677	0.108	−0.628	−6.241	<0.001					
	Territory	−0.021	0.009	−0.183	−2.372	0.018					
	Profession	0.239	0.034	0.560	7.110	<0.001					
	Gender	0.082	0.062	0.074	1.326	0.186					
	Age	0.001	0.008	0.004	0.066	0.947					
CAMI social restriction	(Constant)	2.635	0.355		7.416	<0.001	0.518	0.268	0.254	0.4907	1.696
	QualityRights Training	−0.800	0.118	−0.656	−6.773	<0.001					
	Territory	−0.026	0.010	−0.202	−2.720	0.007					
	Profession	0.310	0.037	0.641	8.457	<0.001					
	Gender	0.124	0.067	0.099	1.836	0.068					
	Age	0.003	0.009	0.021	0.329	0.743					
CAMI mental health ideology	(Constant)	2.891	0.342		8.449	<0.001	0.379	0.144	0.127	0.4726	1.693
	QualityRights training	−0.520	0.114	−0.479	−4.573	<0.001					
	Territory	−0.015	0.009	−0.132	−1.639	0.102					
	Profession	0.202	0.035	0.471	5.737	<0.001					
	Gender	0.089	0.065	0.080	1.363	0.174					
	Age	0.002	0.008	0.018	0.258	0.796					

HRXSW, Human Rights Exposure in Social Work scale; HRESW, Human Rights Engagement in Social Work scale; QRA, World Health Organization’s QualityRights Practices Questionnaire, subscale A; QRB, World Health Organization’s QualityRights Practices Questionnaire, subscale B; CAMI, Community Attitudes Toward the Mentally III scale.

For the HRESW commitment subscale, the model explained a low portion of variance (adjusted *R* ^2^ = 1.2%), with gender being the only significant predictor (*B* = −0.305, *p* = 0.044), indicating lower levels of reported commitment by one gender compared with the other. This finding may appear inconsistent with the lack of significant group differences according to the Mann–Whitney *U*-test (*p* = 0.864). However, the regression analysis controls for other sociodemographic factors, allowing the unique contribution of each variable to be isolated. Therefore, the observed effect of gender likely reflects its independent association with commitment when other predictors are held constant.

In relation to coercive practices, QualityRights training significantly predicted lower scores on the QRA subscale (*B* = −3.118, *p* < 0.001) and CAMI authoritarianism subscale (*B* = −0.853, *p* < 0.001), and higher scores on the QRB subscale (*B* = 2.213, *p* < 0.001), indicating a reduction in coercive or authoritarian tendencies and an increase in more respectful practices as a result of the training. These models showed high explanatory power (adjusted *R* ^2^: QRA = 9.2%; QRB = 89.7%; CAMI authoritarianism = 28.9%). QualityRights training also significantly reduced beliefs related to benevolence (*B* = −0.677, *p* < 0.001), social restriction (*B* = −0.800, *p* < 0.001) and mental health ideology (*B* = −0.520, *p* < 0.001), suggesting a decrease in paternalistic, restrictive or medicalising views regarding mental health.

Across several models, profession was also a significant predictor, particularly for the QRA and QRB subscales, and the CAMI authoritarianism, benevolence, social restriction and mental health ideology, reflecting relevant differences depending on professional role. However, QualityRights training emerged as the most consistent and significant predictor, associated with beneficial changes in reducing authoritarian beliefs and strengthening human rights knowledge.

## Discussion

This study aimed to translate and validate the WHO QualityRights Practices Questionnaire into Spanish for use in Colombian healthcare settings, and to examine the effects of QualityRights training on healthcare professionals’ attitudes, knowledge and coercive practices. Validation yielded excellent psychometric properties (KMO = 0.945; Cronbach’s *α* = 0.891–0.923; 73.4% variance explained by a single component). Both study hypotheses were confirmed: trained professionals demonstrated greater human rights knowledge (*B* = 0.691, *p* = 0.027), lower self-reported coercive practices (*B* = –3.118, *p* < 0.001) and reduced authoritarian attitudes (*B* = –0.853, *p* < 0.001).

Through the QualityRights initiative, the WHO supports countries in implementing policies, strategies, laws and services aligned with international human rights standards, including the CRPD. The internal validity and reliability analysis of the WHO QualityRights Practices Questionnaire revealed a unidimensional structure explaining 73.4% of the total variance, with the instrument demonstrating excellent internal consistency for both subscales (Cronbach’s *α* > 0.89). These results confirm the instrument’s robustness for use in the Colombian healthcare context and support its contribution to monitoring rights-based mental health practices. Given the challenges in monitoring and implementing this strategy in Latin American contexts, the present study proposed a translation and validation of an instrument to monitor coercive practices in healthcare services. This instrument demonstrated psychometric properties like those found in previous validations,^16^ supporting its use in hypothesis testing.

In this regard, both hypotheses were confirmed. Healthcare professionals who received training in QualityRights demonstrated greater awareness of human rights, more favourable perceptions of individuals with mental disorders – particularly in the dimensions of authoritarianism and benevolence – and a lower endorsement of coercive practices. Notably, commitment to human rights did not differ significantly between the two groups, which contrasts with findings from other studies involving students from similar populations, where training improved both commitment and understanding of human rights, as well as other variables related to perceptions of mental disorders, such as mental health ideology.^4^ This may suggest the need for ongoing professional support following the initial training, as recommended by the QualityRights strategy, including continuous guidance and oversight from healthcare service administrators.

Various studies have implemented the QualityRights initiative in different ways, including service evaluations, training delivery and innovations in its application.^21^ In Ghana, the initiative was introduced both in person and online starting in 2016. Regarding this training, Osei et al^22^ reported that 40 443 individuals enrolled in the programme, 25 416 began the training and 20 865 obtained certifications. However, despite this large-scale training effort, changes in coercive practices were not documented.

Similar research, such as that by Poynton-Smith et al,^23^ demonstrated that the online QualityRights training effectively shifted healthcare workers’ attitudes, particularly regarding treatment choice, legal capacity and coercion. In India, a study compared nine public mental health services – six implemented the QualityRights training and three did not. After a 12-month follow-up, services that had adopted the QualityRights training showed significant improvements in attitudes toward people with mental disorders (effect sizes ranging from 0.50 to 0.17).

Additionally, patients reported feeling more empowered and satisfied with the care received (effect size = 0.09).^24^ Other European studies have similarly highlighted the initiative’s effectiveness in improving attitudes, reducing coercive practices and stigma, and promoting independent living among individuals with mental disorders.^25–27^ Other studies have shown the usefulness of QualityRights in hospital settings, and have also pointed to strengthening the dissemination of the strategy through academia,^28^ finding similar challenges in the face of structural stigma and administrative barriers.

In educational settings, many students approve of coercive techniques as part of mental health treatment. For instance, Harden et al^29^ found that 7.91% of students supported the use of restraint and seclusion, and 48.83% believed that involuntary admission was more beneficial than harmful. Potts et al^30^ developed an intervention and indicated that anti-stigma training was associated with positive changes in attitudes, skills and patient-perceived empathy among medical students.

A similar study conducted in Colombia using QualityRights training with medical students found significant differences in stigma, autonomy validation and the perception of social sciences as strategic allies. It also revealed an increased awareness of human rights in relation to individuals with mental health conditions.^4^ These findings support the idea that human rights training should go beyond theoretical content, incorporating practical scenarios to foster lasting commitment. This approach aligns with recommendations to integrate human rights-focused curricula involving individuals with lived experience as expert trainers.^23^ It is worth noting that the QRB subscale captures participants’ perceptions of practices by others. Therefore, an increase in QRB scores after training may reflect heightened critical awareness and improved ability to recognise coercive practices, rather than an actual change in colleagues’ behaviours.

In the Colombian context, the implementation of human rights frameworks in mental health services faces significant structural and regulatory challenges. Although Law 1616 of 2013 recognised the right to mental health, it lacked binding mechanisms to limit coercive practices. More recently, the Statutory Health Law (Law 1751 of 2015) affirmed health as a fundamental right, and the Mental Health Law (Law 2460 of 2025) provided a more robust framework for guaranteeing rights-based care. However, gaps remain in translating these legal advances into institutional practices. The National Mental Health Policy emphasises community-based, person-centred care, but effective monitoring and accountability tools remain limited.^18^ In this context, the WHO QualityRights Practices Questionnaire may serve as a practical tool for assessing service conditions, training healthcare personnel and guiding compliance with national and international human rights standards in mental healthcare delivery.

According to comments made by students who participated in the training, changes in practice require changes at the political or organisational level, with the support of leadership. As individuals learning to practice within the current system, medical students may find it difficult to imagine the possibility of making such changes. Therefore, support from health systems and other protection sectors, in addition to updating legislative elements, are required for more effective implementation of the QualityRights initiative.

This study has several limitations. First, it did not employ a randomised controlled design, as it was part of the evaluation of an educational programme. The comparison was conducted with healthcare teams selected through convenience sampling, although they were representative of the study’s objectives. Another limitation was the decision not to include the additional instruments recommended by the WHO as part of the QualityRights framework,^16^ as the research team had already validated other tools to assess human rights and perceptions of individuals with mental disorders.^4^

Additionally, no pre-training assessment was conducted, which limits the ability to attribute observed changes exclusively to the QualityRights training. The comparison groups were not equivalent: the trained group consisted primarily of medical students, whereas the control group included practising professionals from diverse settings with varying levels of experience. This difference may have influenced baseline attitudes and perceptions. Furthermore, all outcomes – related to attitudes and practices – were measured through self-report instruments, which are subject to social desirability bias and may not accurately reflect actual behaviour. Another limitation was the decision not to include the additional instruments recommended by the WHO as part of the QualityRights framework, as the research team had already validated other tools to assess human rights and perceptions of individuals with mental disorders.

In conclusion, in addition to proposing a translated version of the WHO QualityRights Practices Questionnaire on healthcare practices, this study suggests that implementing the QualityRights initiative among students could reduce the use of coercive practices in clinical settings, lower stigma and improve perceptions of mental disorders, while enhancing understanding of human rights. However, to ensure long-term changes in mental health ideology and sustained commitment to human rights, continuous processes and closer support during implementation are necessary. For future research directions, it would be valuable to explore the medium- and long-term impact of this training on clinical practices, attitudes, and service transformation across diverse healthcare settings, as highlighted in prior evaluations.

## Supporting information

10.1192/bjo.2026.11042.sm001 Agudelo-Hernández et al. supplementary material 1 Agudelo-Hernández et al. supplementary material

10.1192/bjo.2026.11042.sm002 Agudelo-Hernández et al. supplementary material 2 Agudelo-Hernández et al. supplementary material

## Data Availability

The data-sets used and/or analysed during the current study are available from the corresponding author, F.A.-H., on reasonable request.

## Supplementary material

The supplementary material is available online at https://doi.org/10.1192/bjo.2026.11042
