# Supplementary material for: QualityRights in medical education to reduce coercion in mental health in Colombia: instrument validation and quasi-experimental study
Source: BJPsych Open. 2026 May 11;12(3):e132. doi: 10.1192/bjo.2026.11042 (PMC13169050; doi:10.1192/bjo.2026.11042)
Supplement: Agudelo-Hernández et al. supplementary material 1 — Agudelo-Hernández et al. supplementary material [file S2056472426110424sup001.docx]

**Supplement 1. Training protocol.**

A structured training protocol based on the **WHO QualityRights e-training** was implemented for **194 final-year medical students** during their psychiatry training. The course was delivered in **Spanish**, through the official **self-guided virtual platform** of the World Health Organization, and was incorporated as a compulsory academic activity within the final semester curriculum. Although completion of the training was mandatory as part of the course, participation in the research study and completion of study questionnaires were voluntary. The training was carried out over a **six-week period**, with **one session or module per week**, following a predefined sequence of contents and academic accompaniment.

- During **week 1**, students completed an introductory session focused on the **Universal Declaration of Human Rights**, stigma, and the relevance of a human rights-based approach to mental health care. This initial stage aimed to provide a conceptual framework for the rest of the training and to introduce the principles underlying the WHO QualityRights initiative.
- During **week 2**, students completed the module **Human Rights, Mental Health and Disability**, which addressed the rights of persons with mental health conditions and psychosocial disabilities, discrimination in care settings, and the implications of rights violations in clinical practice.
- During **week 3**, students completed the module **Legal Capacity and the Right to Decide**, focused on supported decision-making, autonomy, and respect for the will and preferences of service users, with emphasis on alternatives to substitute decision-making in health care.
- During **week 4**, students completed the module **Ending Coercion, Violence and Abuse**, which addressed coercive practices such as seclusion, restraint, forced treatment, and verbal aggression, and introduced rights-based alternatives for the prevention and elimination of such practices in mental health services.
- During **week 5**, students completed the module **Quality Services and Community Inclusion**, which focused on person-centred care, community-based support, social inclusion, and the characteristics of services aligned with recovery and human rights principles.
- During **week 6**, students completed the module **Mental Health, Well-being and Recovery**, which addressed recovery-oriented care, dignity, hope, and the role of health professionals in promoting well-being and social participation.

Although the course was primarily self-directed, students received **academic support from a psychiatry instructor** during the rotation in order to clarify concepts and contextualize the contents within clinical training. In addition, the training included accompaniment from **a person with lived experience of coercive practices**, who contributed a service-user perspective and supported reflection on coercion, rights violations, recovery, and the importance of person-centred care. This component was intended to strengthen the experiential and reflective dimension of the training and to complement the online modules with direct exposure to lived experience.

**Training completion** was documented through the **issuance and submission of completion certificates** generated by the WHO QualityRights platform after students had completed the assigned modules. Certificates were used only as evidence of adherence to the educational activity and not as a measure of learning outcomes. **Training outcomes** were assessed through study instruments administered in Spanish, which examined attitudes toward people with mental illness, human rights exposure and engagement, and self-reported practices related to coercion.

Specifically, participants completed an ad hoc form for sociodemographic variables, the **Community Attitudes Towards the Mentally Ill (CAMI)** scale, the **Human Rights Exposure in Social Work (HRXSW)** scale, the **Human Rights Engagement in Social Work (HRESW)** scale, and the **WHO QualityRights Practices Questionnaire – Coercion subscale**, including both self-reported use of coercive practices and perceptions of coercive practices among other professionals in their institution. Thus, the certificates verified training completion, whereas the questionnaires were used to evaluate variables potentially associated with the training.
