# Supplementary material for: QualityRights in medical education to reduce coercion in mental health in Colombia: instrument validation and quasi-experimental study
Source: BJPsych Open. 2026 May 11;12(3):e132. doi: 10.1192/bjo.2026.11042 (PMC13169050; doi:10.1192/bjo.2026.11042)
Supplement: Agudelo-Hernández et al. supplementary material 2 — Agudelo-Hernández et al. supplementary material [file S2056472426110424sup002.docx]

| Supplement 2. Traduction World Health Organization’s QualityRights Practices Questionnaire.  Por favor indique cuantas veces en los últimos tres meses ha usado las siguientes estrategias dentro de su unidad psiquiátrica | | | | | | | | |
| --- | --- | --- | --- | --- | --- | --- | --- | --- |
|  | | **7Todos los días** | **6Algunas veces a la semana** | **5Una vez a la semana** | **4Algunas veces al mes** | **3Una vez al mes o menos** | **2Algunas veces en los últimos 3 meses** | **1 Nunca** |
| QR1 | **Use la reclusión/aislamiento** |  |  |  |  |  |  |  |
|  | (por ejemplo, ordenar u mantener a un usuario en una habitación cerrada) |  |  |  |  |  |  |  |
| QR2 | **Use restricciones físicas** |  |  |  |  |  |  |  |
|  | (por ejemplo, usar correas u otros dispositivos mecánicos para sujetar a los usuarios) |  |  |  |  |  |  |  |
| QR3 | **Prescribí o administré un tratamiento, aunque el usuario no lo quería** |  |  |  |  |  |  |  |
| QR4 | **Use restricciones químicas** |  |  |  |  |  |  |  |
|  | (por ejemplo, prescribí o administré una inyección para calmar el comportamiento del usuario sin su consentimiento) |  |  |  |  |  |  |  |
| QR5 | G**rite o use agresión verbal para que los usuarios cumplieran las solicitudes** |  |  |  |  |  |  |  |
| Para cada enunciado, marque la opción que refleje con mayor precisión su respuesta.(INVERSA) | | | | | | | | |
|  | | **1Mucho menos que yo** | | **2Menos que yo** | | **3Igual que yo** | **4Mas que yo** | **5Mucho más que yo** |
| QRB1 | Los profesionales de salud mental en mi unidad usan la reclusión/el aislamiento y las restricciones físicas/químicas |  | |  | |  |  |  |
| QRB2 | Los profesionales de salud mental en mi unidad gritan o usan la agresión verbal para que los usuarios cumplan las solicitudes |  | |  | |  |  |  |
| QRB3 | Los profesionales de salud mental en mi unidad prescriben o administran tratamientos para controlar la conducta de los usuarios. |  | |  | |  |  |  |
| QRB4 | Los profesionales de salud mental en mi unidad usan restricciones para controlar situaciones de agitación e sala |  | |  | |  |  |  |
